# Identification and Characterization of *CYP9A40* from the Tobacco Cutworm Moth (*Spodoptera litura*), a Cytochrome *P450* Gene Induced by Plant Allelochemicals and Insecticides

**DOI:** 10.3390/ijms160922606

**Published:** 2015-09-18

**Authors:** Rui-Long Wang, Christian Staehelin, Qing-Qing Xia, Yi-Juan Su, Ren-Sen Zeng

**Affiliations:** 1 Key Laboratory of Tropical Agro-Environment, Ministry of Agriculture, College of Natural Resources and Environment, South China Agricultural University, Guangzhou 510642, China; E-Mails: rlw2009@scau.edu.cn (R.-L.W.); qingqingxiaqq@gmail.com (Q.-Q.X.); syj@scau.edu.cn (Y.-J.S.); 2 Key Laboratory of Agroecology and Rural Environment of Guangdong Regular Higher Education Institutions, South China Agricultural University, Guangzhou 510642, China; 3 State Key Laboratory of Biocontrol and Guangdong Key Laboratory of Plant Resources, School of Life Sciences, Sun Yat-sen University (East Campus), Guangzhou 510006, China; E-Mail: cst@mail.sysu.edu.cn; 4 College of Life Sciences, Fujian Agriculture and Forestry University, Fuzhou 350002, China

**Keywords:** *Spodoptera litura*, cytochrome P450 monooxygenase, RNA interference, insecticide detoxification, plant allelochemicals

## Abstract

Cytochrome P450 monooxygenases (P450s) of insects play crucial roles in the metabolism of endogenous and dietary compounds. Tobacco cutworm moth (*Spodoptera litura*), an important agricultural pest, causes severe yield losses in many crops. In this study, we identified *CYP9A40*, a novel *P450* gene of *S. litura*, and investigated its expression profile and potential role in detoxification of plant allelochemicals and insecticides. The cDNA contains an open reading frame encoding 529 amino acid residues. *CYP9A40* transcripts were found to be accumulated during various development stages of *S. litura* and were highest in fifth and sixth instar larvae. *CYP9A40* was mainly expressed in the midgut and fat body. Larval consumption of xenobiotics, namely plant allelochemicals (quercetin and cinnamic acid) and insecticides (deltamethrin and methoxyfenozide) induced accumulation of *CYP9A40* transcripts in the midgut and fat body. Injection of ds*CYP9A40* (silencing of *CYP9A40* by RNA interference) significantly increased the susceptibility of *S. litura* larvae to the tested plant allelochemicals and insecticides. These results indicate that *CYP9A40* expression in *S. litura* is related to consumption of xenobiotics and suggest that CYP9A40 is involved in detoxification of these compounds.

## 1. Introduction

Cytochrome P450 monooxygenases (P450s or CYPs) represent a large enzyme family, with representatives in most organisms [1]. P450s of insects can metabolize and thereby detoxify insecticides and plant allelochemicals [2,3,4]. Insect P450s have been divided into four major clades: CYP2, CYP3, CYP4, and mitochondrial P450s [1]. The most common P450s belong to the CYP3 clade (CYP3, CYP6, CYP9 members). Like other P450s, CYP3 clade enzymes may have important functions in detoxification of xenobiotics and plant allelochemicals [1,4].

The polyphagous tobacco cutworm moth (*Spodoptera litura* Fabricius) is a well-known pest that causes severe yield losses of many important crops such as soybean, tomato, potato, cotton and groundnut [5]. At present, many field populations of this pest have developed high resistance against various insecticides [5]. Pest control of *S. litura* has become increasingly difficult all over the world, particularly in many Asian countries [6]. Outbreaks of *S. litura* are mainly attributed to its ability to adapt to a wide array of host plants and development of resistance to commonly applied insecticides [5,6]. *S. litura* can tolerate in its diet considerable amounts of plant allelochemicals or pesticides [5,7]. Quercetin is a typical plant allelochemical produced by many host plants frequently attacked by *S. litura*, while cinnamic acid is rarely encountered by *S. litura* larvae [4]. By a practical point of view, quercetin has a good potential for use as biocontrol agent against *S. litura* larvae*.* Deltamethrin [7] is a pyrethroid (class II) and methoxyfenozide [8] is a diacylhydrazine insecticide. For *S. litura* control, deltamethrin and methoxyfenozide are mainly used as larvicidal or adulticidal agents [5,6]. Deltamethrin and methoxyfenozide show strong contact and stomach toxicity for insects [7,8].

In insects, gene silencing through RNA interference (RNAi) is a powerful tool to silence target genes, e.g., genes required for insect development and insect-plant relationships [9]. Silencing of lepidopteran genes by RNAi has been successfully performed by delivery of double-stranded RNA (dsRNA) through microinjection, ingestion or soaking [10].

To better understand the ability of *S. litura* to tolerate plant allelochemicals and insecticides, we became interested in the role of *P450* genes of this pest [4,6]. In this study, we isolated and characterized a novel *P450* gene from *S. litura*, named *CYP9A40.* We investigated the expression of *CYP9A40* during different developmental stages and in different tissues. We also explored the function of *CYP9A40* by using RNAi-silenced larvae that were fed with diets containing plant allelochemicals or insecticides. The results suggest a role of *CYP9A40* in detoxification of plant allelochemicals and insecticides.

## 2. Results

### 2.1. Identification of CYP9A40

**Figure 1:**
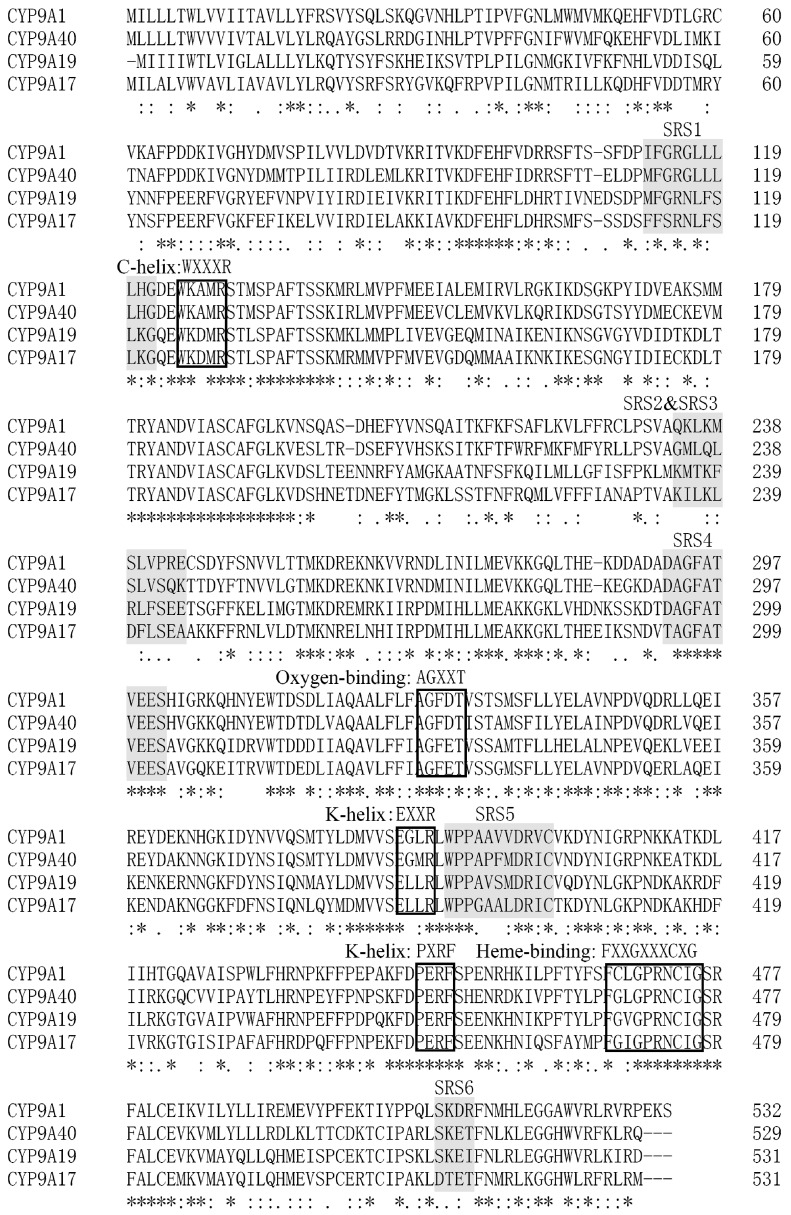
Alignment of the amino acid sequences deduced from *CYP9A40* (*Spodoptera litura*) with *CYP9A1* (*Heliothis virescens*), *CYP9A17* (*Helicoverpa armigera*) and *CYP9A19* (*Bombyx mori*). Predicted substrate recognition sites (SRSs) are highlighted. Conserved motifs (WXXXR, AGXXT, EXXR, PXRF and FXXGXXXCXG) are also marked on the boxes. Conserved amino acid residues are indicated below: “*” means a single, fully conserved residue; “:” indicates a strongly and “.” a weakly conserved residue.

*CYP9A40*, a cytochrome *P450* gene of *S. litura*, was cloned. The full-length 1639-bp cDNA sequence of *CYP9A40* contains a 38 bp 5′-untranslated region (5′-UTR), an open reading frame (ORF) of 1590 bp, and an 11-bp 3′-untranslated region (3′-UTR). The sequence was deposed at the GeneBank database (accession number: KR065418). The ORF encodes a predicted protein of 529 amino acids. CYP9A40 has a theoretical pI value of 8.57 and a predicted molecular mass of 61.48 kDa. CYP9A40 contains the signature motif of P450s and shares highest amino acid sequence identity with members of the CYP9A subfamily [11,12,13]. By aligning *CYP9A40* with three CYP9A subfamily members from *Helicoverpa* (Figure 1), it was found that they share several conserved motifs [12,13], namely the C-helix sequence WXXXR (WKAMR at position 125–129 of CYP9A40), the K-helix motif EXXRXXP (EGMRLWP at position 385–391 of CYP9A40), the heme-binding decapeptide motif FXXGXXXCXG (FGLGPRNCIG at position 466–475 of CYP9A40), the oxygen-binding motif AGXXT (AGFDT at position 327–331 of CYP9A40), and the putative “meander”-binding sequences EXXR and PXRF (EGMR at position 385–388; PERF at positions 447–450 of CYP9A40)*.* Based on sequence similarities, six putative substrate recognition sites (SRSs) [12] were also identified in CYP9A40 (Figure 1).

**Figure 2:**
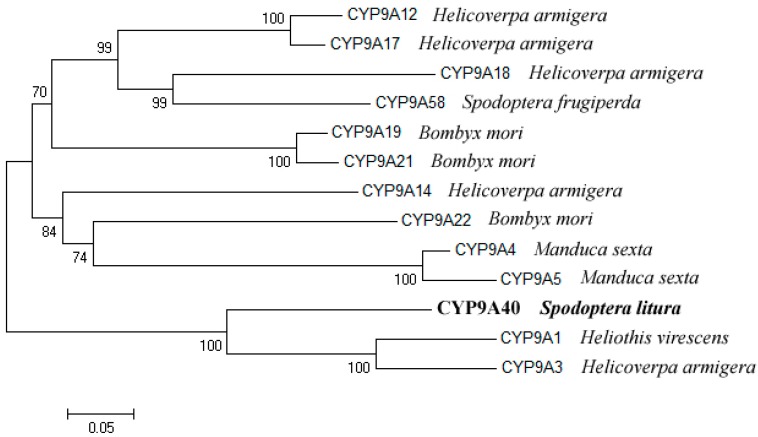
Phylogenetic analysis of *CYP9A40* of *S. litura* and related P450s from various insects. The phylogenetic tree was constructed from generated alignments using the neighbor-joining (NJ) method of the Mega 4.0 software (MEGA, Tempe, AZ, USA). The values on the branches indicate the percentage frequencies at which the phylogram topology was representative for 1000 bootstrap replicates. The scale bar indicates 0.05 amino acid substitutions per site.

A phylogenetic tree was built with CYP9A subfamily amino acid sequences from various Lepidoptera. The analysis showed that CYP9A40 is most related to CYP9A1 of *Heliothis virescens*. CYP9A40 and CYP9A1 have an amino acid identity of 70.7%. The amino acid identities of CYP9A40 with CYP9A3 of *Helicoverpa armigera* and CYP9A58 of *Spodoptera frugiperda* are 69.4% and 56.4%, respectively (Figure 2).

### 2.2. Accumulation of CYP9A40 Transcripts during Different Developmental Stages

**Figure 3:**
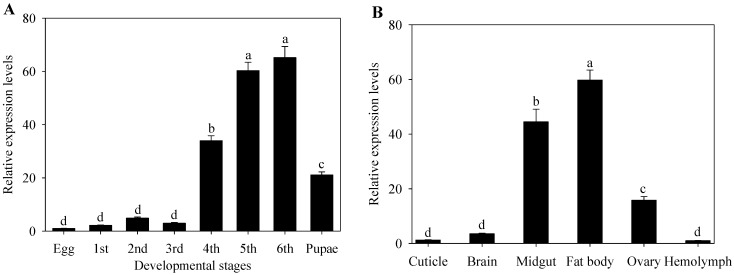
Gene expression levels of *S. litura* CYP9A40 at different development stages (**A**) and in different tissues (**B**) relative to that in eggs and hemolymph respectively as determined by qRT-PCR analysis. Data shown are means ± SE. Different letters above bars indicate significant differences (*p* < 0.05) according to one-way ANOVA followed by the Duncan’s multiple range test.

qRT-PCRs were conducted to determine the expression pattern of *CYP9A40* during various developmental stages of *S. litura* (Figure 3A). Transcripts of *CYP9A40* were detected for all samples. Low transcript levels of *CYP9A40* were measured from the egg to the third instar larval stage. Transcripts of *CYP9A40* strongly accumulated in fourth (34-fold higher than in eggs), fifth (60-fold higher than in eggs) and sixth (65-fold higher than in eggs) instar larvae. Expression of *CYP9A40* was significantly higher in fifth and sixth instar larvae than in the other developmental stages. Transcripts of *CYP9A40* were also elevated in pupae (21-fold higher than in eggs).

### 2.3. Expression of CYP9A40 in Different Tissues

We further investigated expression levels of *CYP9A40* in different tissues. RNAs were extracted from cuticle, brain, midgut, fat body, ovary and hemolymph dissected from sixth instar larvae (Figure 3B). qRT-PCR analysis revealed that *CYP9A40* transcripts could be detected in all examined tissues. The results showed that the larvae accumulated high levels of *CYP9A40* transcripts in the fat body (60-fold higher than in hemolymph), midgut (44-fold higher than in hemolymph) and ovary (16-fold higher than in hemolymph). In contrast, gene expression of *CYP9A40* was found to be relatively low in the cuticle, brain and hemolymph.

### 2.4. Induction of CYP9A40 Expression by Plant Allelochemicals and Insecticides

**Figure 4:**
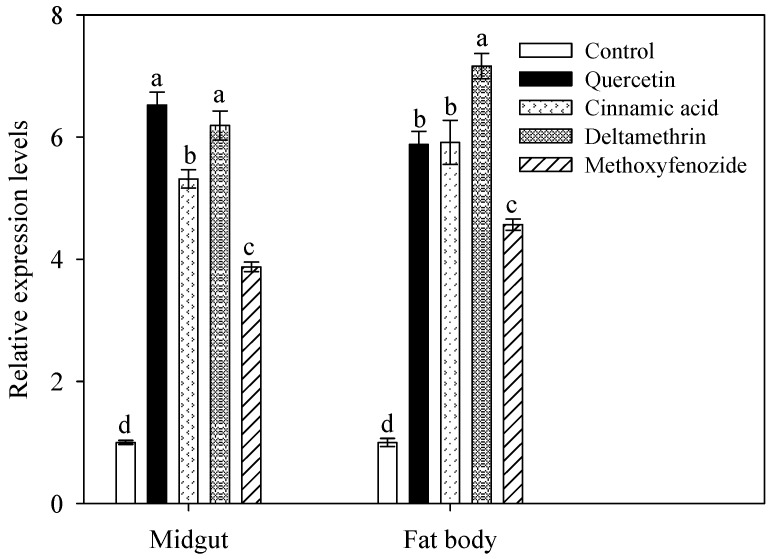
Effect of quercetin, cinnamic acid, deltamethrin and methoxyfenozide on the accumulation of *CYP9A40* transcripts in the midgut of fifth instar larvae in response to uptake of plant allelochemicals and insecticides for 48 h. Control larvae were fed on artificial diet without xenobiotic compounds. Transcript levels were determined by qRT-PCR. Data shown are means ± SE. Different letters above bars indicate significant differences (*p* < 0.05) according to one-way ANOVA followed by the Duncan’s multiple range test.

To determine the effect of plant allelochemicals and insecticides on accumulation of *CYP9A40* transcripts, qRT-PCR was performed using 2-day old fifth instar larvae fed for 48 h with artificial diet supplemented with quercetin, cinnamic acid, deltamethrin and methoxyfenozide. Compared to control larvae (fed with artificial diet without xenobiotics), *CYP9A40* expression in the midgut significantly increased in response to uptake of quercetin (6.5-fold), cinnamic acid (5.3-fold), deltamethrin (6.2-fold) and methoxyfenozide (3.9-fold) (Figure 4). A similar increase in *CYP9A40* expression was seen in samples from the fat body (5.8-fold induction for quercetin, 5.9-fold for cinnamic acid, 7.2-fold for deltamethrin and 4.6-fold for methoxyfenozide) (Figure 4). Hence, up-regulation of *CYP9A40* expression in the midgut and fat body of *S. litura* was observed in response to consumption of all four tested xenobiotics.

### 2.5. Silencing of CYP9A40 by RNAi Suggests a Role of CYP9A40 in Detoxification of Plant Allelochemicals and Insecticides

**Figure 5:**
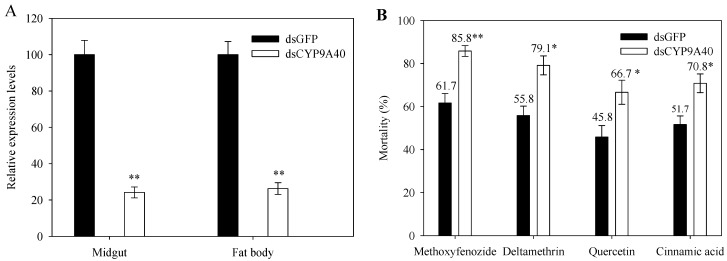
Changes in the susceptibility of fifth instar larvae to different xenobiotics after injection of dsCYP9A40. Control larvae were subjected to injection with the same amounts of dsGFP. Data shown are means ± SE obtained from four biological repeats. (**A**) qRT-PCR analysis of *CYP9A40* transcript levels 24 h after delivery of dsCYP9A40 and dsGFP, respectively. Expression of *CYP9A40* was considerably silenced by RNAi as marked by two asterisks (Student’s *t*-test, *p* < 0.01); (**B**) Effects of uptake of plant allelochemicals and insecticides on mortality of larvae. Larvae were fed on artificial diet supplemented with indicated xenobiotics for 24 h. Mortality of larvae injected with dsCYP9A40 was elevated in response to all four xenobiotics. Asterisks above bars indicate a significant increase in mortality of dsCYP9A40-injected larvae compared to those injected with dsGFP (Student’s *t*-test, * *p* < 0.05, ** *p* < 0.01).

To explore a possible function of CYP9A40 in detoxification of plant allelochemicals and insecticides, *CYP9A40* expression in fifth instar larvae was silenced by an RNAi approach. Microinjection was performed with dsCYP9A40 and dsGFP served as a control. Expression analysis of *CYP9A40* by qRT-PCR was conducted 24 h after delivery of dsRNA. Compared to control larvae, larvae injected with dsCYP9A40 showed significantly lower levels of *CYP9A40* transcripts (decrease to 24.2% in the midgut and to 26.3% in the fat body). These results indicate considerable silencing of *CYP9A40* by RNAi (Figure 5A).

We further used the *CYP9A40-*silenced larvae to examine their susceptibility to plant allelochemicals and insecticides. Larvae injected with dsCYP9A40 or dsGFP were fed with artificial diet supplemented with a given xenobiotic and their survival was assessed 24 h later. When exposed to the tested xenobiotics, the *CYP9A40-*silenced larvae showed increased mortality rates as compared to the dsGFP injected control larvae. Delivery of dsCYP9A40 (compared to dsGFP) increased larval mortality caused by cinnamic acid (from 51.7% to 70.8%) and quercetin (from 45.8% to 66.7%). Similar changes were also observed for deltamethrin (increase from 55.8% to 79.1%) and methoxyfenozide (from 61.7% to 85.8%) (Figure 5B). These findings suggest that CYP9A40 is implicated in detoxification of plant allelochemicals and insecticides.

## 3. Discussion

Herbivorous insects have evolved a variety of mechanisms to adapt to allelochemicals of host plants and insecticides. Adaptation includes production of detoxifying enzymes such as P450s, glutathione *S*-transferase and carboxylesterases [14,15]. P450s are well known for their roles in metabolism and detoxification of plant allelochemicals and insecticides [1]. The polyphagous pest *S. litura* encounters large quantities of plant allelochemicals and insecticides. In this study, we cloned and characterized the *P450* gene *CYP9A40* of *S. litura*. The amino acid sequence of CYP9A40 contains various motifs conserved in P450s including predicted SRSs (Figure 1). SRS1, SRS4 and SRS5 are highly conserved within the CYP9 subfamily [12]. CYP9A40 shares 70.7%, 63.8% and 54.4% amino acid identity with CYP9A1 of *H. virescens*, CYP9A58 of *S. frugiperda* and CYP9A17 of *H. armigera*, respectively (Figure 2). CYP9A1 of *H. virescens* is the prototype of the CYP9A subfamily and expression of this gene in tobacco budworms was associated with a reduced susceptibility to the carbamate insecticide thiodicarb [11]. *CYP9A58* of *S. frugiperda* was 2.7-fold increased in the midgut when sixth instar larvae were exposed to methoxyfenozide [15,16]. Expression of *CYP9A17* was increased in the midgut and particularly in the fat body (14.5-fold), when sixth-instar larvae of *H. armigera* were exposed to the *P450* gene inducer phenobarbital [12].

Expression pattern of inducible genes can provide useful information on their biological functions [13,17]. To characterize expression of *CYP9A40* in *S. litura*, we determined expression levels of *CYP9A40* at different development stages and in different tissues. Highest transcript levels were found in fourth to sixth instar larvae, particularly in the midgut and fat body (Figure 3). The midgut and fat body of insects are important tissues associated with detoxification of xenobiotics [4,13,18]. Like for *CYP9A40*, transcript levels of other *P450* genes of *S. litura*, namely *CYP6B47*, *CYP6B48* and *CYP6B58*, were strongly accumulated in the larval midgut and fat body [4,19]. The expression pattern of *CYP9A40* is also reminiscent to that of *CYP9A61*, a *P540* gene of *Cydia pomonella.* Strongest expression of *CYP9A61* was found in fourth to sixth instar larvae and levels of *CYP9A61* transcripts in the fat body were found to be 384.0-fold higher than in the cuticle. However, *CYP9A61* expression in the midgut was relatively low (10-fold increase compared to the cuticle) [13].

Various studies have shown that the P450 detoxification system in insects can be induced by a broad range of xenobiotic compounds [2]. In the silkworm (*Bombyx mori*), for example, the activity of P450s in the midgut was 2.3-fold stimulated after feeding larvae with diet supplemented with quercetin [20]. In *H. armigera* exposed to fenvalerate (a type II synthetic pyrethroid) for 14 generations, transcripts of *CYP9A12*, *CYP9A14*, and *CYP6B7* showed strongly induced levels in the fat body of larvae [21]. Heterologous expression of *CYP9A12* and *CYP9A14* in yeast suggests that these P450s of *H. armigera* can play role in detoxification of insecticides such as esfenvalerate (the (*S*)-enantiomer of fenvalerate) [22]. Expression of *CYP6AE11*, another *P450* gene of *H. armigera*, was found to be strongly expressed in pyrethroid resistant strains and it was suggested that CYP6AE11 also plays a role in detoxification of xenobiotics [23]. Moreover, transcripts of the *CYP6B6* gene were found to be strongly accumulated in the midgut and fat body of *H. armigera* and expression of this gene was stimulated in a feeding experiment with plant allelochemicals (2-tridecanone and quercetin). Transcript levels of this gene correlated with the concentration of applied 2-tridecanone but not with the quercetin concentration [24]. In *Helicoverpa zea*, expression of four *CYP6B* genes (*CYP6B8*, *CYP6B9*, *CYP6B27* and *CYP6B28*) was increased in response to plant allelochemicals, namely coumarin, quercetin, xanthotoxin, and flavone [25]. CYP6B8 and CYP321A1 of *H. zea* have been demonstrated to metabolize insecticides (including aldrin, cypermethrin and diazinon) as well as plants allelochemicals such as xanthotoxin [26]. In *S. litura*, expression levels of *CYP6B58* in the midgut were slightly enhanced by uptake of xanthotoxin (1.7-fold) or coumarin (1.5-fold) [4]. CYP6B58 and other P450s of *S. litura*, namely CYP6B48 and CYP6AB14, likely metabolize plant allelochemicals such as cinnamic acid, quercetin, coumarin, xanthotoxin and flavone [4,27]. In the present feeding study, uptake of quercetin, cinnamic acid, deltamethrin and methoxyfenozide could significantly stimulate expression of *CYP9A40*, indicating up-regulated gene expression by a broad range of xenobiotics (Figure 4). The observed differences in *CYP9A40* up-regulation may be due to differences in consumption, cellular uptake, recognition and detoxification of these xenobiotics.

RNAi is a powerful method to assess the roles of *P450* genes in detoxification of plant allelochemicals and insecticides [2,27,28]. In this study, microinjection of dsCYP9A40 into *S. litura* larvae resulted in a noticeable reduction of *CYP9A40* transcripts in the midgut and fat body. Remarkably, injection of dsCYP9A40 followed by feeding with quercetin, cinnamic acid, deltamethrin or methoxyfenozide significantly increased mortality rates of the instar larvae (Figure 5B). These results indicate that silencing of *CYP9A40* by RNAi could increase the susceptibility to the tested plant allelochemicals and insecticides. However, it cannot be completely excluded that microinjection of dsCYP9A40 also caused a slight reduction of transcript levels of other *P450* genes of *S. litura.* Nevertheless, the results of our feeding experiment provide first clues that CYP9A40 is a novel *P450* gene that can detoxify plant allelochemicals and insecticides. CYP9A40 likely acts synergistically with other P450s of *S. litura*. In fact, silencing of the *CYP6AB14* gene by RNAi followed by feeding with plant allelochemicals (xanthotoxin, coumarin or flavone) also resulted in increased mortality rates of *S. litura* larvae [27]. These results are reminiscent to RNAi-mediated silencing of *P450* genes in other insects. In *H. armigera* for example, silencing of *CYP6AE14* by RNAi significantly reduced the growth of larvae that were fed with cotton (*Gossypium hirsutum* cv. R15) [29]. Likewise, silencing of *CYP321E1* in the moth *Plutella xylostella* resulted in increased mortality of fourth-instar larvae exposed to the insecticide chlorantraniliprole [30]. Furthermore, reduction of *CYP9AQ2* expression by RNAi led to an increased mortality of third-instar nymphs of *Locusta migratoria* that were exposed to the insecticide deltamethrin [28]. How monooxygenase activity of CYP9A40 contributes to detoxification of different xenobiotics in *S. litura* cells should be analyzed in further studies.

In summary, we identified and characterized *CYP9A40*, a novel *P450* gene of the moth *S. litura*. *CYP940* expression varied with developmental stages and tissues. It was highest in the midgut and fat body of sixth instars. Uptake of plant allelochemicals and insecticides stimulated expression of *CYP9A40* in larvae. Silencing of *CYP9A40* in larvae led to increased larval susceptibility to these compounds, suggesting an important role of CYP9A40 in detoxification of xenobiotics. Future work is required to characterize the CYP9A40 protein and to examine synergistic effects with other P450s of *S. litura.*

## 4. Experimental Section

### 4.1. Insects

Larvae of *S. litura* were provided by the Insectarium of the Institute of Entomology, Sun Yat-sen University (Guangzhou, China) and maintained in an insectary without exposure to any insecticides and chemicals at South China Agricultural University (Guangzhou, China). Larvae of *S. litura* were feed on artificial diet [4,19] and were reared at 25 ± 2 °C and 70% ± 5% relative humidity under a 16-h light/8-h dark photoperiod.

### 4.2. Cloning of the CYP9A40 Gene

CYP9A subfamily member-like nucleotide sequences were identified by a Blast similarity search (BLASTN and TBLASTN algorithm; available online: http://blast.ncbi.nlm.nih.gov) using expressed sequence tag (EST) sequences derived from RNA isolated from the midgut of sixth instar larvae of *S. litura* [31]. Reverse transcription-polymerase chain reaction (RT-PCR) using gene-specific primers derived from an EST contig sequence (CYP9A40-F: 5′-ATGCTGCTGTTACTGACGTGGG-3′; CYP9A40-R: 5′-CTGGCGCAGCTTAAACCTGACC-3′) was employed to obtain the full-length cDNA sequence of the *CYP9A40* gene (coding sequence and untranslated regions). The amplification conditions were as follows: 95 °C for 2 min, 35 cycles (95 °C for 45 s, 55 °C for 50 s and 72 °C for 90 s) and a final extension step of 72 °C for 10 min. The PCR product was ligated into the pGEM-T Easy Vector (Promega Inc., Beijing, China) and sequenced using an ABI 377 capillary automated sequencer. The open reading frame (ORF) of *CYP9A40* was obtained by the ORF Finder program at the NCBI homepage (Available online: http://www.ncbi.nlm.nih.gov/gorf/gorf.html).

### 4.3. RNA Extraction and cDNA Synthesis

Total RNA was extracted using TRIzol (Invitrogen, Carlsbad, CA, USA) according to the manufacture’s instructions. The quality and concentration of RNA were examined by agarose gel electrophoresis and spectrophotometer analysis. Extracted RNAs were treated with DNase I (Invitrogen) and then reverse transcribed using the ThermoScript™ RT-PCR System kit (Life Technologies, Grand Island, NY, USA) according to the instructions of the provider.

### 4.4. Phylogenetic Analysis

Phylogenetic analysis was performed for 12 amino acid sequences, which were considered to be representative for lepidopteran P450s involved in metabolism of xenobiotics: CYP9A40 from *S. litura*, CYP9A1 from *H. virescens* (tobacco budworm), CYP9A4 and CYP9A5 from *Manduca sexta* (goliath worm), CYP9A3, CYP9A12, CYP9A14, CYP9A17 and CYP9A18 from *H. armigera* (cotton bollworm), CYP9A19, CYP9A21 and CYP9A22 from *B. mori* (silkworm) and CYP9A58 from *S. frugiperda* (fall armyworm). The phylogenetic tree was built using Mega 4.0 software (MEGA, Tempe, AZ, USA) [32] and the neighbor-joining method. The inferred phylogeny was tested by bootstrap analysis with 1000 replicates. The alignments, with amino acid substitutions labeled as non-conservative, weakly conservative or strongly conservative, were generated with the ClustalX program software [33]. Substrate recognition sites (SRSs) [12] in these proteins were predicted by alignment with the amino acid sequences of CYP9A1 of *H. virescens* and CYP9A17 of *H. armigera* as described by Zhou *et al*. [12].

### 4.5. Quantitative Real-Time PCR

Relative expression levels of *CYP9A40* were determined by quantitative real-time RT-PCR (qRT-PCR). The gene-specific primers CYP9A40F (5′-TACAGGCTTCTACCATCCG-3′) and CYP9A40R (5′-TGGCGTCATACTCCCTAAT-3′) were used for detection of *CYP9A40* transcripts. The *β-actin* gene was used as an internal standard gene to normalize the expression levels among different samples. The primers of the *β-actin* gene (*β-actin* F: 5′-TGAGACCTTCAACTCCCCCG-3′; *β-actin* R: 5′-GCGACCAGCCAAGTCCAGAC-3′) have been used before [21]. Real-time PCR reactions were performed using an MJ Research Opticon instrument (Bio-rad Inc., Hercules, CA, USA) in a volume of 20 μL containing 10 μL of 2 SYBR Green I Master Mix (Roche Diagnostics Corp., Indianapolis, IN, USA), 0.2 M of each primer and 1 μL cDNA template. The PCR conditions were as follows: 95 °C for 5 min, 35 cycles of 95 °C for 45 s, 64 °C for 45 s and 72 °C for 1 min. To assess the specificity of PCR amplifications, a dissociation-curve analysis was performed at the end of the runs. The relative expression levels of *CYP9A40* were determined by using the 2^−ΔΔ*C*t^ method [34].

### 4.6. Expression of CYP9A40 during Different Developmental Stages and in Various Tissues

For analyses of the *CYP9A40* expression pattern at different development stages of *S. litura*, RNA was extracted from eggs (30 eggs per RNA extraction), first to sixth instar larvae at day 2 (4 larvae per RNA extraction) and pupae at day 2 (3 pupae per RNA extraction). For analysis of the *CYP9A40* expression pattern in different tissue types, RNA was extracted from cuticle, brain midgut, fat body, ovary and hemolymph from fifth instar larvae. RNA isolation and qRT-PCR were done as described above. Three independent RNA extractions were performed for each analyzed biological material.

### 4.7. Expression Analysis of CYP9A40 in Response to Xenobiotics

Newly molted fifth instar larvae of *S. litura* were fed for 48 h on artificial medium [4,19] containing different doses of xenobiotics (plant allelochemicals and insecticides): 1% (*w*/*w*) quercetin (QUE, ≥98%, Sigma-Aldrich, St. Louis, MO, USA), 1% (*w*/*w*) cinnamic acid (CIN, ≥98%, Sigma-Aldrich, St. Louis, MO, USA), 0.05% (*w*/*w*) deltamethrin (DEL, 99%, Bayer CropScience, Monheim, Germany) and 0.03% (*w*/*w*) methoxyfenozide (MET, 24%, Dow AgroSciences LLC, Indianapolis, IN, USA). After 48 h, both midgut and fat body were dissected from surviving larvae. The material was then immediately frozen in liquid nitrogen and kept at −80 °C for RNA extraction and further *CYP9A40* expression analysis. Twenty individuals were used for each treatment. Three independent RNA extractions (representing three biological replicates) were performed for all treatments.

### 4.8. Silencing of CYP9A40 by RNAi and Bioassay with Xenobiotics

An RNAi approach was used to silence *CYP9A40* expression in fifth instar larvae. The *CYP9A40* cDNA templates for *in vitro* transcription reactions were prepared by PCR using cDNA (derived from RNA of 4 fifth instar larvae) as template. Two pairs of primers (dsCYP9A40 F (5′-AATACGACTCACTATAGGGTACAGGCTTCTACCATCCG-3′) and CYP9A40 R (5′-TGGCGTCATACTCCCTAAT-3′) (nucleotide position 1069–1087 in the ORF), CYP9A40 F (5′-TACAGGCTTCTACCATCCG-3′) (nucleotide position 676–694 in the ORF) and dsCYP9A40 R (5′-AATACGACTCACTATAGGGTGGCGTCATACTCCCTAAT-3′)) were used to synthesize dsRNA for *CYP9A40* (676–1087 bp of ORF), which contained the T7 promoter region in both sense and antisense strands. The primers were designed based on the sequence of *CYP9A40* and the amplification conditions were 94 °C for 5 min, 35 cycles (94 °C for 30 s, 55 °C for 30 s and 72 °C for 1 min) and a final extension step (72 °C for 10 min). As a control, dsGFP (688 bp, GenBank accession ACY56286) was synthesized by the same PCR method using the following primer pairs: T7GFPdsRNA F (5′-AATACGACTCACTATAGGGAAGGGCGAGGAGCTGTTCACCG-3′) and GFPdsRNA R (5′-CAGCAGGACCATGTGATCGCGC-3′); GFPdsRNA F (5′-AAGGGCGAGGAGCTGTTCACCG-3′) and T7GFPdsRNA R (5′-AATACGACTCACTATAGGGCAGCAGGACCATGTGATCGCGC-3′) [27,35]. PCR products were purified using the QIAquick PCR Purification Kit (Qiagen Inc., Valencia, CA, USA), and DNA concentrations were determined using a spectrophotometer. DsRNA corresponding to *CYP9A40* (dsCYP9A40) and *GFP* (dsGFP) were synthesized using a T7 RiboMAX™ Express RNAi System (Promega, Madison, WI, USA) according to the manufacturer’s instructions. The final concentrations of dsCYP9A40 and dsGFP were adjusted to 1.5 µg·µL^−1^ by DEPC-treated (RNase-free) water and kept at −80 °C. For all dsRNA injection experiments, fifth instar (day 2) larvae were used and 2 µL (3.0 µg) of dsRNA were injected into the side of the thorax of each larva using a manual microinjector (model No. MS05, Chengdu Centome Company Ltd., Chengdu, China). The injection points were sealed immediately with paraffin [27]. After injection, the larvae of *S. litura* were kept at 25 ± 2 °C, 70% ± 5% relative humidity and a 16-h light/8-h dark photoperiod.

Expression levels of *CYP9A40* were analyzed 24 h after the injection of dsCYP9A40 or dsGFP. RNA extraction and expression analysis of *CYP9A40* by qRT-PCR were performed as described above. In total, 32 fifth instar (day 2) larvae were used for the *CYP9A40* expression analysis. Each extracted RNA sample corresponded to four larvae (4 independent biological replica per treatment).

For bioassays with plant allelochemicals and insecticides, microinjections of dsCYP9A40 or dsGFP were done for totally 240 fifth instar (day 2) larvae, which were randomly assigned into two groups before microinjection (120 each). The larvae were injected with 2 µL of dsCYP9A40 or dsGFP as described above. After dsRNA delivery, the larvae from each group were assigned into four subgroups (30 each), and individuals in each subgroup were kept on artificial diets containing plant allelochemicals or insecticides at concentrations described above*.* After 24 h, mortality rates (ratio of dead larvae to the sum of tested larvae) were recorded for each subgroup (*n* = 4). The bioassays were performed in triplicate.

### 4.9. Statistical Analysis

Data were expressed as means ± standard error (SE). Statistical analysis was carried out with the SPSS 10.0 software package (IBM Corp., Armonk, New York, NY, USA). One-way ANOVA followed by the Duncan’s multiple range test was employed to analyze differences among different tissues and development stages. The Students’s *t*-test was used to analyze data from the RNAi and feeding experiments with different xenobiotics. Statistical differences were considered significant at *p* < 0.05.
